# Untargeted Metabolomics Insights into Newborns with Congenital Zika Infection

**DOI:** 10.3390/pathogens10040468

**Published:** 2021-04-13

**Authors:** Estéfane da C. Nunes, Ana M. B. de Filippis, Taiane do E. S. Pereira, Nieli R. da C. Faria, Álvaro Salgado, Cleiton S. Santos, Teresa C. P. X. Carvalho, Juan I. Calcagno, Flávia L. L. Chalhoub, David Brown, Marta Giovanetti, Luiz C. J. Alcantara, Fernanda K. Barreto, Isadora C. de Siqueira, Gisele A. B. Canuto

**Affiliations:** 1 Departamento de Química Analítica, Instituto de Química, Universidade Federal da Bahia, Rua Barao de Jeremoabo, 147, Salvador, BA 40170-115, Brazil; estefane.cruz@gmail.com (E.d.C.N.); taianepereira94@gmail.com (T.d.E.S.P.); 2 Laboratório de Flavivírus, Instituto Oswaldo Cruz, Fiocruz, Avenida Brasil, 4365, Manguinhos, Rio de Janeiro, RJ 21040-900, Brazil; ana.bispo@ioc.fiocruz.br (A.M.B.d.F.); flaviallevy@yahoo.com.br (F.L.L.C.); David.Brown@phe.gov.uk (D.B.); giovanetti.marta@gmail.com (M.G.); luiz.alcantara@ioc.fiocruz.br (L.C.J.A.); 3 Laboratório de Genética Celular e Molecular, Universidade Federal de Minas Gerais, Avenida Presidente Antônio Carlos, 6627, Belo Horizonte, MG 31270-901, Brazil; alvaro.salgado.81@gmail.com; 4 Instituto Gonçalo Moniz, Fiocruz, Rua Waldemar Falcão, 121, Salvador, BA 40295-010, Brazil; Cleitonsantos29@outlook.com; 5 Maternidade de Referência Professor José Maria de Magalhães Neto, SESAB, Rua Marquês de Maricá, Salvador, BA 40310-000, Brazil; terezaxpaim@gmail.com (T.C.P.X.C.); calcagnoji@gmail.com (J.I.C.); 6 Instituto Multidisciplinar em Saúde, Universidade Federal da Bahia, Rua Hormindo Barros, 58, Vitória da Conquista, BA 45029-094, Brazil

**Keywords:** Zika virus, microcephaly, metabolomics, GC-MS, machine learning

## Abstract

Zika virus (ZIKV), an emerging virus belonging to the Flaviviridae family, causes severe neurological clinical complications and has been associated with Guillain-Barré syndrome, fetal abnormalities known collectively as congenital Zika syndrome, and microcephaly. Studies have shown that ZIKV infection can alter cellular metabolism, directly affecting neural development. Brain growth requires controlled cellular metabolism, which is essential for cell proliferation and maturation. However, little is known regarding the metabolic profile of ZIKV-infected newborns and possible associations related to microcephaly. Furthering the understanding surrounding underlying mechanisms is essential to developing personalized treatments for affected individuals. Thus, metabolomics, the study of the metabolites produced by or modified in an organism, constitutes a valuable approach in the study of complex diseases. Here, 26 serum samples from ZIKV-positive newborns with or without microcephaly, as well as controls, were analyzed using an untargeted metabolomics approach involving gas chromatography–mass spectrometry (GC-MS). Significant alterations in essential and non-essential amino acids, as well as carbohydrates (including aldohexoses, such as glucose or mannose) and their derivatives (urea and pyruvic acid), were observed in the metabolic profiles analyzed. Our results provide insight into relevant metabolic processes in patients with ZIKV and microcephaly.

## 1. Introduction

Zika virus (ZIKV) was first identified in 1947 in the Zika forest of Uganda by a team monitoring yellow fever. In 1952, human viral infection was initially observed in Uganda, followed by the United Republic of Tanzania [1] and French Polynesia [2]. ZIKV infection was considered benign until October 2015, when an increasing number of newborns born with microcephaly was reported at maternity services in Northeast Brazil [3].

ZIKV is known to be neurotropic [4]. Associations between the infection and neurological disorders as well-established, including ocular and auditory abnormalities [5,6], Guillain-Barré syndrome in adults, and microcephaly in newborns [7]. Most children whose mothers were exposed and experienced viral symptoms during pregnancy present an average head circumference at birth. Children may express neurological manifestations, including delayed neuro-psycho-motor development, and can develop microcephaly after a few months of life [8]. The characterization of factors that contribute to neurocognitive deficits in children, even in the absence of evident anatomical malformations, and the estimation of risk of neurocognitive dysfunction, may have important and substantial clinical and public health implications [9].

Studies have demonstrated that not all newborns with congenital Zika syndrome present microcephaly at birth. Some may have brain abnormalities, such as calcifications, hydrocephalus, ophthalmic changes or postnatal microcephaly, and exhibit delayed neuropsychomotor development at varying degrees of intensity [10]. Due to variability in presentation and the consequences of infection during pregnancy, studies on the metabolic changes induced by ZIKV are recommended to further understand the disease and aid in the development of personalized therapies for affected patients.

Metabolomics is the comprehensive analysis of metabolites on a molecular level, reflecting an organism’s phenotype [11]. Metabolite determination is usually performed by mass spectrometry (MS) coupled to separation techniques. Gas chromatography–mass spectrometry (GC-MS) has become an interesting analytical technique to perform metabolomics studies. GC-MS provides robust metabolic coverage, as this technique detects relatively polar metabolites, such as organic acids, amino acids and sugars [12].

Metabolomics has been scarcely utilized to evaluate metabolic changes in ZIKV infection [13,14,15,16]. A recent systematic review presented theoretical aspects and described works involving metabolomics in the study of arboviruses, including ZIKV [17]. Some of these studies investigated the mechanism of viral replication in adult human serum [14], and one proposed a new machine learning (ML) algorithm for diagnostic purposes [15]. Simultaneously, Diop et al. studied congenital Zika syndrome in microglial cells to investigate the neuroinflammation induced by this infection. These authors found several nonpolar metabolites (lipid derivatives) related to neuronal differentiation, viral replication and apoptosis regulation [16]. Despite demonstrating promise, the use of metabolomics remains limited in the context of ZIKV infection.

The present study applied a metabolomics approach in serum samples from ZIKV-infected newborns to elucidate metabolic changes possibly associated with the microcephaly development.

## 2. Results

### 2.1. Clinical Data and ZIKV Diagnosis

Serum samples were collected from 26 newborns with symptoms suggestive of ZIKV, all born in 2016 at a maternity hospital located in Salvador, Bahia-Brazil. The samples were divided into three groups: confirmed Zika virus infection with microcephaly (ZPMP), ZIKV without microcephaly (ZPMN) and controls (ZNMN). Table S1 (Supplementary Materials) describes all study participants (newborns and mothers), including additional information on ZIKV diagnosis, intergrowth criteria classifications and comorbidities. ZIKV diagnosis was performed by anti-ZIKV immunoglobulin M-IgM or real-time reverse transcription–polymerase chain reaction (rRT-PCR). Microcephaly was determined using intergrowth-21st classification criteria. While the red reflex test was not carried out in all 26 newborns selected for this study, significant alterations were not detected in those individuals submitted to ophthalmological testing. Serological analyses for dengue and chikungunya infections, diseases with similar clinical presentations between patients, were performed and showed no evidence of recent infections in the mothers.

### 2.2. Metabolomics Study

The effects of ZIKV infection and microcephaly were investigated via an untargeted metabolomics approach using GC-MS analysis. Serum samples were randomly analyzed after protein precipitation and derivatization using oximation and silylation reactions. Quality controls (QC) were prepared by pooling equal volumes of all samples used to verify instrumental stability during analysis. After data acquisition, XCMS [18] software was used to extract molecular features (MFs), entities characterized by an *m*/*z* signal intensity or area, and retention time (RT). Before MF extraction, RT alignment, data filtering and grouping were performed according to the parameters described in the Materials and Methods section. The data matrix was normalized utilizing median values and statistically evaluated using the MetaboAnalyst platform [19]. Figure S1 (Supplementary Materials) details the Principal Component Analysis (PCA) scores plot and Partial Least-Squares Discriminant Analysis (PLS-DA) plots, derived from models built using normalized data from the three evaluated groups (ZPMP, ZPMN and ZNMN). The supervised method revealed clear distinctions among the groups, demonstrating differentiation between metabolic profiles.

Metabolites were annotated based on a built-in library (Fiehn RT Library) [20] after data deconvolution, correlation of retention index values and retention time correction using AMDIS (Automated Mass Spectral Deconvolution and Identification System) software. Twenty metabolites and chemical classes were assigned based on retention time and mass spectra fragmentation patterns.

**Figure 1:**
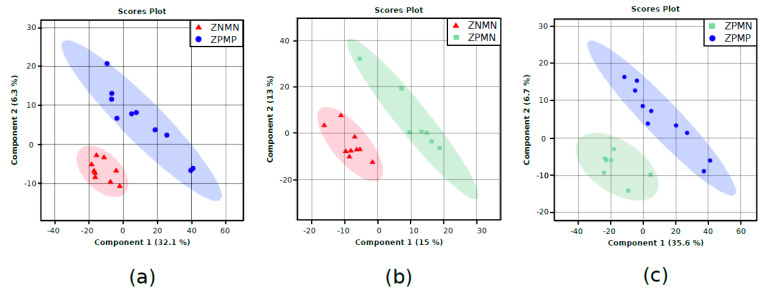
Scores plots for PLS-DA models built in MetaboAnalyst platform [19] using GC-MS median normalized data sets, log-transformation and pareto scaling. (**a**) ZPMP vs. ZNMN, (**b**) ZPMN vs. ZNMN and (**c**) ZPMP vs. ZPMN. Group samples: ZPMP (*n* = 10), zika virus with microcephaly (blue dots); ZPMN (*n* = 7), zika virus without microcephaly (green squares); and ZNMN (*n* = 9), control samples (red triangles).

To investigate the effects of ZIKV infection and microcephaly on metabolic profile, multivariate and univariate methods were employed to find discriminants. Figure 1 illustrates PLS-DA models detailing metabolic changes corresponding to infection in conjunction with microcephaly ZPMP versus ZNMN (Figure 1a), ZIKV infection alone (comparing ZPMN versus ZNMN; Figure 1b) and microcephaly alone (comparing ZPMP versus ZPMN; Figure 1c).

The PLS-DA models shown in Figure 1 demonstrate excellent differentiation between groups. Significant MFs were identified using Variable Importance Projection (VIP) scores by selecting entities with a VIP score > 1.0. A univariate statistical method using ANOVA followed by post-hoc testing (Tukey’s, *p*-value < 0.05) was applied to complement the search for discriminants. Statistical significance was verified in 12 metabolites and chemical classes through multivariate and univariate analyses. Among these, important changes were observed in the levels of amino acids and carbohydrates. Table 1 presents the fold changes of discriminating metabolites identified in our analyses, with results divided according to chemical class. FC (fold change) was calculated by the ratio of the first group to the second group. FC > 1.0 means that the metabolite is up-regulated, while FC < 1.0 means downregulation. For example, in DL-isoleucine, FC = 0.97 for ZPMP vs. ZNMN, which means that the metabolite is decreased in the ZPMP group compared to ZNMN.

Concerning metabolite annotation, it is important to emphasize that the GC-MS analytical technique is limited in its capability to distinguish among some isomers, such as L-threonine and L-allothreonine, which have very similar retention times (RTs) and mass spectral fragmentation patterns. Another difficulty arises in differentiating among some metabolites classified as carbohydrates and their derivatives. In this case, the most abundant characteristic fragments and derivatized products are almost identical, and annotation is therefore limited to the hemiacetal conformation of these sugars [12].

Machine learning methods were used to complement the multivariate and univariate analyses. Three XGBoost (extreme gradient boosting) classifiers models were applied to the three comparisons studied (ZPMP vs. ZNMN, ZPMN vs. ZNMN, and ZPMP vs. ZPMN), with subsequent model interpretation using SHAP (SHapley Additive exPlanations) methodology, in order to identify further significant metabolite changes in each comparison. ML models were adjusted with 50% of the data used for training and validation, while the remaining 50% was utilized for testing. Figure S2 (Supplementary Materials) illustrates the influence of metabolites on group classification for SHAP value graphic and ROC curves for the three comparisons. Under SHAP analysis, each metabolite is classified according to its effect (positive or negative) in each respective comparison. The ROC curves attest to the performance of the ML model. For ZPMP vs. ZNMN, methyl-beta-D-galactopyranoside, hexose (tagatose/L-sorbose)/hexosamine (D-lyxosylamine) and phosphoric acid were more upregulated in control samples (ZNMN). In the ZIKV infection comparison (ZPMN vs. ZNMN), urea, phosphoric acid, L-serine, L-threonine/L-allothreonine, glucoheptonic acid/lactones (ribonic acid-gamma-lactone or gluconic acid lactone), stearic acid and methyl-beta-D-galactopyranoside were all found to be downregulated in infection. In the microcephaly comparison (ZPMP vs. ZPMN), urea, glucoheptonic acid/lactones and pyruvic acid were upregulated, while N-methylglutamic acid was downregulated in microcephalic newborns. The results obtained from the machine learning method were compared to those obtained by multivariate analysis. Some of the metabolite variations were identified by both approaches, reinforcing these results. Others were identified by each method separately. Table 1 shows details on each significant metabolite identified on the three comparisons. Further details concerning the statistical results (VIP score and *p*-value), chemical characteristics and fold change (FC) values calculated for each group comparison are presented in Table S2 (Supplementary Materials). It is important to emphasize that we consider a molecular feature as statistically significant when either one of the methods of the analysis point to significance according to the cut-off of VIP score > 1.0, *p*-value < 0.05 and “high feature value” for the model’s classification method SHAP analysis of the ML approach (Figure S2). Fold changes drive a differentiation between comparisons and were calculated by the ratio between the median intensities of first by second compared groups for each metabolite. FC values less than unit means that the metabolite is downregulated in the first group compared to the second. On the other hand, FC > 1.0 indicates an increase in the first group. As the larger the differentiation of the FC from the unit, the greater the metabolite impact on the studied system. Biologically, the determination of FC points to the variation of concentration of the metabolites, indicating an increase or decrease in the production of a given metabolite in a pathway depending on the condition studied. FC determination is important for understanding and raising hypotheses of the complex metabolic alterations caused by the studied condition [11]. At this stage, we cannot provide information about the biological impact of the FC magnitude. However, we believe that these variations can result in the accumulation of the metabolite by inhibiting specific enzymes, important cellular alterations for viral maintenance, or replication, resulting in a deficiency of key-metabolites and high energy expenditure. Certainly, our preliminary findings need further investigation.

**Figure 2:**
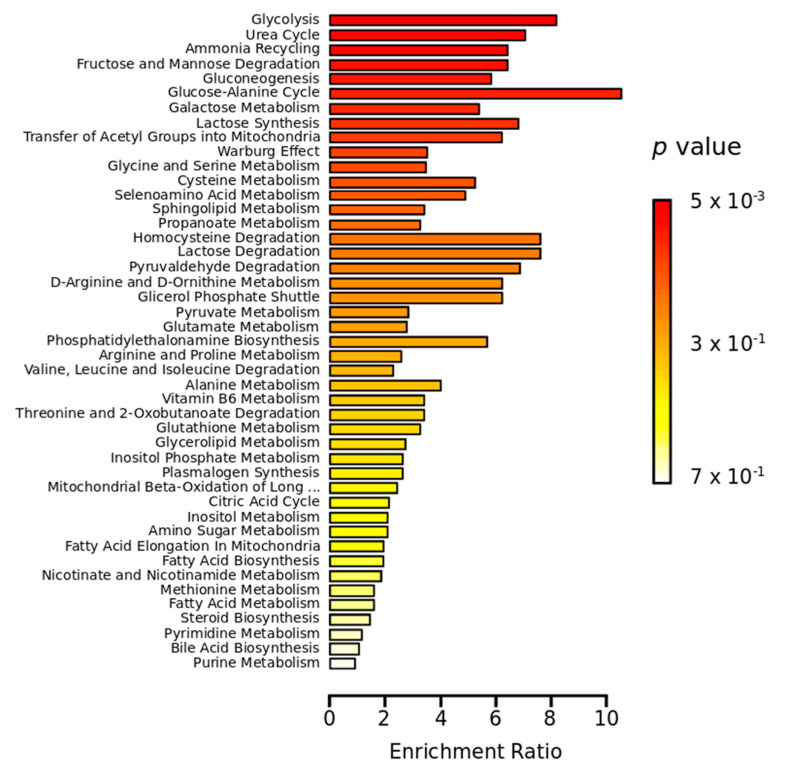
Metabolite enrichment analysis. Red horizontal bars summarize the most significantly altered metabolic pathways.

Overall, significant downregulation (FC less than unit) of amino acids and derivatives, carbohydrates and conjugates, and organic and fatty acids were identified when comparing groups ZPMP and ZPMN to the control group (ZNMN). In the assessment of microcephaly associated with ZIKV infection (ZPMP vs. ZPMN), significant increases (FC up to 1.21) in amino acids (L-serine and L-threonine/L-allothreonine), glucoheptonic acid/lactones, pyruvic acid and urea were observed. In order to further elucidate alterations in metabolic pathways corresponding to these conditions, enrichment analysis was performed using the MetaboAnalyst platform [19]. Figure 2 illustrates that the Glucose-Alanine Cycle, Glycolysis, Urea Cycle, Gluconeogenesis and carbohydrate-associated metabolic pathways were the most significantly altered pathways identified in this analysis.

## 3. Discussion

During the 2015−2016 Zika outbreak in Brazil, it was discovered that ZIKV infection during pregnancy could result in a spectrum of diseases in infants, including congenital disabilities and neurodevelopmental disorders, known as Congenital Zika Syndrome, identified in childhood. Nonetheless, the probable mechanism underlying impairment in brain development and function provoked by this arbovirus remains unclear [21,22]. The present study employed an untargeted metabolomics approach to better characterize the metabolic profile of newborns infected with ZIKV in order to gain deeper insight into the development of microcephaly.

Table 1 and Table S2 (Supplementary Materials) list relevant metabolites belonging to the classes of carbohydrates, organic acids and amino acids, which are found to be discriminant between the compared groups. Our data show decreased levels of aldohexoses (glucose or mannose) in newborns infected with ZIKV compared to control groups (ZNMN), as well as those with microcephaly (ZPMP) compared to infected newborns (ZPMN), as reflected by fold change values of 0.60 and 0.28, respectively (Table S2). As previously mentioned, the GC-MS technique is limited in the differentiation of sugars (such as glucose and mannose). Those metabolites have similar spectral fragmentation patterns and retention times, making it difficult to differentiate accurately. For this reason, we represent the identification by the class of the hemiacetal (aldohexose) in Table 1 and Table S2. The hypotheses presented here for the biological explanation are based on the metabolites described in the literature. These carbohydrate deficiencies may be related to impaired brain development, possibly leading to the outcome of microcephaly. As viruses are dependent on host cells, they use the same regulatory and signaling pathways as the host. In this sense, it has been well established that virus replication processes can modify cellular metabolism through alterations in metabolic pathways [23]. Usually, in eukaryotic cells, glucose is metabolized to pyruvate in a process known as glycolysis. The pyruvate is then converted into acetyl-CoA to generate ATP by oxidative phosphorylation. Oxidative phosphorylation provides significantly more ATP per unit of glucose than glycolysis. However, several independent studies have demonstrated that some viruses could induce glycolytic activation and probably use the ATP produced by glycolysis in host cells to their advantage [24,25,26,27].

Similar to other arboviruses, studies have suggested that ZIKV-induced glycolysis is necessary for viral replication, and that the glycolysis inhibition attenuates this rate [26,28,29]. Regarding the development of microcephaly, it is important to consider that glucose is the primary molecule used by neurons for oxidative phosphorylation and ATP generation and that the presence of glucose is fundamental during fetal growth [30]. It is important to highlight that while controlled cellular metabolism is essential for normal brain development, recent studies have shown that ZIKV directly infects neural stem cells in the fetus, disturbing host cellular metabolism [31,32]. If the virus can induce glycolytic activation to the extent that insufficient glucose levels become available for brain development, malformations may occur. Our metabolite enrichment analysis pointed to that glycolysis was one of the most significantly altered pathways in both ZIKV infection alone as well as in microcephalic newborns (Figure 2). Our results further demonstrate that newborns with microcephaly present lower aldohexose levels (glucose or mannose) than those without this abnormality, despite both groups being infected by ZIKV (ZPMP vs. ZPMN). This finding implies that the microcephaly outcome is likely multifactorial in nature, possibly due to genetic factors related to the host or genetic variations in viral strains. We also observed increased pyruvic acid (FC 1.31, Table S2), a product of glycolysis, in the ZPMP group compared to ZPMN, which further corroborates the importance of glucose, i.e., if ZIKV-induced glycolysis occurs, it is expected that the metabolic product of this reaction would be found in excess. Our results also suggest differences in glucose (aldohexose) levels when comparing ZPMP to ZNMN, which supports the notion that this carbohydrate is essential for normal brain development.

Among documented ZIKV complications, microcephaly and ocular malformations are the most relevant abnormalities [33,34]. Several studies on ZIKV-associated congenital eye malformations have reported retinal degeneration, bilateral macular and perimacular lesions, and optic nerve abnormalities. However, due to deficient knowledge surrounding the possible mechanisms involved in this pathology, it remains unclear whether ocular lesions are a direct result of ZIKV infection or related to the development of microcephaly itself [35,36,37,38]. In this sense, sorbitol (sugar alcohol) could be a molecule that is key to better understanding the pathogenesis of ZIKV-associated congenital eye malformations. This metabolite has been associated with retinopathy development. It is known that excessive glucose levels can lead to the metabolization of this monosaccharide into fructose through sorbitol and that increased intracellular accumulation of sorbitol can cause a hyperosmotic effect that results in diabetic cataract formation [39]. Here we verify that, in addition to glucose, newborns with microcephaly present lower levels of sorbitol (sugar alcohol) than those without microcephaly, despite both being infected by ZIKV (FC 0.43). Our findings lead us to suggest that since microcephalic newborns have less sorbitol available, the development of ocular lesions during childhood would not likely be associated with the accumulation of this metabolite. It is important to highlight that it was not possible to investigate ocular findings in this study thoroughly. Therefore, we suggest more in-depth ophthalmological evaluations in affected patients to further investigate this hypothesis.

In an effort to better understand molecular disorders and microcephaly development associations, we also evaluated levels of amino acids and derivatives in the studied newborns. Amino acids are molecules that serve as building blocks, provide sources of energy and exert biological functions in specific tissues, such as the brain. Each amino acid has two enantiomeric forms, denoted L- and D-amino acids. In mammals, L-amino acids predominate, although some D-amino acids are also involved in cellular functions [40]. As expected, we observed significantly elevated L-serine levels in the microcephalic newborns with ZIKV infection (FC 1.21, ZPMP vs. ZPMN). Serine is a non-essential amino acid synthesized from intermediates involved in the glycolytic pathway. Since the glycolytic pathway is overactivated in ZPMP, increases in this amino acid are an expected finding.

Interestingly, excessive L-serine can be converted to D-serine, which acts as a co-agonist of the N-methyl-d-aspartate receptor subtype of glutamate receptors (NMDAr), which regulates neural function [41]. As the proper functioning of this receptor is required by neuronal networks for efficient brain activity and cognition-related abilities, increased L-serine levels in patients with microcephaly are remarkable.

Importantly, our analysis of ZPMN vs. ZNMN suggested significantly reduced DL-isoleucine, L-serine, L-threonine/L-allothreonine and L-valine in ZIKV-infected newborns. Decreased levels of urea were also observed in these newborns. Since nitrogen produced as a byproduct of amino acid catabolism is detoxified in the liver via the urea cycle, lower urea levels would be expected in patients with reduced levels of isoleucine, serine, threonine and valine, such as newborns infected with ZIKV.

Many questions remain unanswered concerning ZIKV and its teratogenic effects on the fetal brain. Identifying changes in the metabolic profile of infants exposed to ZIKV with and without microcephaly may provide valuable insight into the molecular mechanisms underlying this neurological disorder. Our results suggest relevant alterations in the metabolic profile of ZIKV-infected newborns who develop microcephaly. It is important to emphasize that the biological interpretations described here are hypotheses, based on a pilot study and extensive literature search, and require further investigation. The use of other omic approaches (lipidomics, proteomics, genomics) and metabolomics using complementary analytical platforms, both applied to a new set of samples, could validate and add new information to our findings. There are few studies on metabolic changes resulting from ZIKV infection and consequent microcephaly. We hope that our findings provide new insights and aid future studies focused on disease comprehension and therapeutic applications.

## 4. Materials and Methods

### 4.1. Ethics Statement

This study was approved by the Institutional Review Board of the Gonçalo Muniz Institute, Oswaldo Cruz Foundation (IGM-FIOCRUZ, protocol no. 1.935.854/2016) and the Flavivirus Laboratory, IOC/FIOCRUZ, Rio de Janeiro, Brazil (CAAE: 90249218.6.1001.5248). The legal guardians of all included newborns provided a signed term of informed consent.

### 4.2. Studied Groups and Sample Collection

Participants were recruited from a previous neonatal surveillance program for Congenital Zika Infection in 2016 at a public maternity hospital in the municipality of Salvador (Bahia), located in Northeastern Brazil [42]. All enrolled newborns were classified according to the criteria established by the International Fetal and Newborn Growth Consortium for the 21st Century (INTERGROWTH-21st). Microcephaly was defined as a head circumference below two standard deviations from the mean for sex and gestational age. A typical head circumference was considered when measurements were within two standard deviations from the mean [43]. Biological samples were obtained from umbilical cord blood collected at birth.

The participants were divided into three groups: controls, *n* = 9 (denominated ZNMN); zika virus without microcephaly, *n* = 7 (ZPMN); and zika virus with microcephaly, *n* = 10 (ZPMP).

### 4.3. ZIKV Diagnosis

All serum samples were collected at birth from the umbilical cord vein. Ethylenediaminetetraacetic acid (EDTA) plasma was obtained by centrifugation and stored at −80 °C until analysis. Serological and molecular ZIKV diagnoses were performed according to previously described methods [42,44]. Congenital Zika was defined when newborns presented positivity upon serological testing (anti-ZIKV immunoglobulin M-IgM) or real-time reverse transcription PCR (rRT-PCR) for ZIKV. Healthy controls had negative serological and molecular results for ZIKV. In all samples, testing for syphilis, HIV, Toxoplasma (IgM), and Cytomegalovirus (IgM) returned negative results. The red reflex test was conducted at the maternity hospital to detect ophthalmological abnormalities in newborns.

### 4.4. Metabolomics Sample Preparation

Serum samples from newborns were thawed in an ice bath and homogenized by vortexing (Phoenix, AP56, Garbsen, Germany). Aliquots of 100 µL of each sample were mixed with cold isopropanol (1:3, *v*/*v*, sample/solvent) to ensure protein precipitation. The samples were maintained for 60 min at −20 °C, then centrifuged for 10 min at 13,500× *g*, after which 200 µL of supernatant was transferred to GC insert vials. Quality control samples (QC) were prepared by pooling equal volumes (70 µL) of all studied samples. A blank sample was prepared using deionized water instead of serum and submitted to identical protein precipitation procedures. Serum, QC and blank samples were evaporated to dryness using SpeedVac equipment (Martin Christ, model RVC 2-18 CDplus, Vienna, Austria) at 35 °C. Dried samples were derivatized prior to GC-MS analysis in two steps: (i) oximation was performed by adding 20 µL *O*-methoxyamine (15 mg mL^−1^) in pyridine, followed by incubation at room temperature for 90 min; (ii) silylation by adding 20 µL *N*,*O*-bis(trimethylsilyl)trifluoroacetamide with 1% trimethylchlorosilane (*v*/*v*), after which samples were placed in an oven for 30 min at 40 °C. Derivatized samples were then resuspended in 120 µL of heptane, homogenized and analyzed by GC-MS.

### 4.5. GC-MS Metabolomics Analysis

GC-MS analyses were performed in a gas chromatograph (7820 A, Agilent Technologies, Santa Clara, CA, USA) hyphenated to a single quadrupole mass spectrometer (5977E MSD, Agilent Technologies, CA, USA). Separations were performed in a DB5-MS column (60 m length, 0.32 mm internal diameter, 0.25 μm film 95% dimethylpolysiloxane/5% diphenyl polysiloxane, Agilent Technologies). The approach applied herein was adapted from a previously described method [45]. Briefly, He was used as carrier gas at a flow rate of 1.0 mL min^−1^. The injector temperature was maintained at 250 °C. Samples were run at a 1:10 split ratio using He at 10 mL min^−1^. The temperature gradient was programmed at 50 °C, with an increasing ramp rate of 5 °C min^−1^ up to 325 °C. The MS parameters utilized were transfer line, filament source and quadrupole at 290, 230 and 150 °C, respectively. The electron ionization source used −70 eV energy, and the MS was operated in scan mode in the range of 50–600 *m*/*z*. MassHunter Qualitative Analysis B.07.01 (Agilent Technologies) software was used for instrument operation and data acquisition.

### 4.6. Metabolomics Data Processing and Statistical Analysis

Raw data were converted into mzML format using ProteoWizard 3.0 software and processed using the XCMS package (version 3.6.1) executed in R (version 3.6.0). The data matrix was extracted using the “matchedFilter” method with the following parameters: full width at half maximum (fwhm = 4), signal-to-noise cutoff (snthresh = 1.5), the maximum number of groups to identify in a single *m*/*z* slice (max = 100), bandwidth (bw = 4 and 2 for first and second grouping, respectively), width of overlapping *m*/*z* slices (mzwid = 0.25) and the minimum fraction of samples necessary in the class to be absent/present (minfrac = 0.5). Alignment was performed using the “retcor” method with non-linear alignment and degree of smoothing for local polynomial regression fitting (span = 0.5). The FillPeaks function was applied to reduce missing values.

Statistical multivariate and enrichment analyses were performed using the MetaboAnalyst 4.0 platform (www.metaboanalyst.ca accessed on 5 August 2020) [19]. Raw data were normalized according to median values. Log-transformation and pareto scaling was applied. Multivariate analyses, including Principal Component Analysis (PCA) and Partial Least-Squares Discriminant Analysis (PLS-DA), were performed to verify instrumental performance and to find discriminants between compared groups (Variable Importance Projection, VIP score > 1.0). One-way ANOVA testing was applied to identify significant alterations among metabolites between the ZPMP, ZPMN and ZNMN groups, followed by Tukey’s post-hoc test (*p*-value < 0.05) (Excel, Microsoft).

### 4.7. Metabolite Identification

Peak detection and deconvolution were performed in the Automated Mass Spectral Deconvolution and Identification System (AMDIS 2.73). Metabolites were identified based on spectra fragmentation patterns, retention index and retention time correction using the Fiehn RT Library [20]. Retention time correction used minimum match factor of 60, reverse mode, RT window of 0.05 min and component width equal 15. To confirm peak assignment a match factor of 85 was selected. Table S2 (Supplementary Materials) presents *m*/*z* qualifier and RT for metabolite assignment.

### 4.8. Machine Learning Analysis

Machine learning analyses were performed using the XGBoost classification algorithm [46,47,48]. Modeling parameters were adjusted using a k-fold cross-validation scheme, and performance was evaluated on a test dataset. Model results were interpreted using SHAP (SHapley Additive exPlanations) framework [49].

## Figures and Tables

**Table 1:** Fold change (FC) values of significant annotated chemical classes and metabolites identified in three comparisons of zika virus infection and microcephaly in newborns.

Chemical Class	Metabolite	ZPMP vs. ZNMN	ZPMN vs. ZNMN	ZPMP vs. ZPMN
Amino acids and derivatives	DL-isoleucine		0.97	
	L-serine		0.71 ^(b)^	1.21
	L-threonine/L-allothreonine ^§^		0.75 ^(b)^	1.37
	L-valine		0.88	
	*N*-methylglutamic acid			0.72 ^(a)^
Carbohydrates and conjugates	Aldohexose (D-glucose/D-mannose) *	0.60		0.28
	Sugar alcohol (D-mannitol/D-sorbitol) *			0.43
	Aldohexose (D-mannose/D-altrose) *	0.61	2.30	0.26
	Glucoheptonic acid/ribonic acid-gamma-lactone/gluconic acid lactone		0.88 ^(a)^	1.67 ^(b)^
	Methyl-beta-D-galactopyranoside	0.52 ^(a)^	0.84 ^(a)^	0.62
	Hexose (tagatose/L-sorbose) */D-lyxosylamine (hexosamine)	0.88 ^(a)^		
Fatty acids and conjugates	Palmitic acid	0.62		
	Stearic acid	0.64	0.84 ^(a)^	
Inorganic acids	Phosphoric acid	0.75 ^(a)^	0.69 ^(a)^	
Organic acids and derivatives	Pyruvic acid			1.31 ^(a)^
	Urea	0.50	0.41 ^(b)^	1.21 ^(a)^

Legend: ZNMN, control group; ZPMN, zika virus without microcephaly group, and ZPMP, zika virus with microcephaly. ^§^ Indistinguishable isomers. * Metabolites indistinguishable by spectral pattern and retention time under GC-MS analysis. Metabolites with FC values > 1.0 are up-regulated and FC < 1.0 are down-regulated in each individual group comparison. ^(a)^ Metabolite/class significative by Machine Learning (ML) alone. ^(b)^ Metabolite/class significative by univariate or multivariate analysis and ML.

## Data Availability

The data presented in this study are available on request from the corresponding author. The data are not publicly available due to lack of financial funding for this purpose.

## Supplementary Materials

The following are available online at https://www.mdpi.com/article/10.3390/pathogens10040468/s1, Figure S1: Multivariate models obtained from serum samples analyzed by GC-MS. (a) Scores plot for PCA model and (b) Scores plot for PLS-DA models. Group samples: ZPMP, zika virus with microcephaly (blue dots); ZPMN, zika virus without microcephaly (green squares); ZNMN, control (red triangles); Figure S2: ZHAP analysis showing the influence of the metabolites for group classification. (a) ZPMP vs. ZNMN, (b) ZPMN vs. ZNMN, and (c) ZPMP vs. ZPMN. Group samples: ZPMP, zika virus with microcephaly; ZPMN, zika virus without microcephaly; ZNMN, control; Table S1: Clinical patients’ description and ZIKV diagnosis results; Table S2: Statistical results of metabolites or classes and chemical characteristics found by untargeted metabolomics of serum samples by GC-MS analysis.
