# Supplementary material for: Untargeted Metabolomics Insights into Newborns with Congenital Zika Infection
Source: Pathogens. 2021 Apr 13;10(4):468. doi: 10.3390/pathogens10040468 (PMC8070065; doi:10.3390/pathogens10040468)
Supplement: Supplementary file 1 [file pathogens-10-00468-s001.zip › pathogens-1135369 supplementary/Figure-S1.docx]

**SUPPLEMENTARY MATERIALS – FIGURE S1**

**Untargeted metabolomics insights of newborns with congenital Zika infection**

Estéfane da C. Nunes, Ana M. B. de Filippis on behalf of ZikAction Consortium, Taiane do E. S. Pereira, Nieli R. da C. Faria, Álvaro Salgado, Cleiton S. Santos, Teresa C. P. X. Carvalho, Juan I. Calcagno Flávia L. L. Chalhoub, David Brown, Marta Giovanetti, Luiz C. J. Alcantara, Fernanda Khouri Barreto, Isadora C. de Siqueira, and Gisele A. B. Canuto.

(a) (b)

**Figure S1.** Multivariate models obtained from serum samples analyzed by GC-MS. (**a**) Scores plot for PCA model and (**b**) Scores plot for PLS-DA models. Group samples: ZPMP, zika virus with microcephaly (blue dots); ZPMN, zika virus without microcephaly (green squares); ZNMN, control (red triangles).
