# Supplementary material for: Untargeted Metabolomics Insights into Newborns with Congenital Zika Infection
Source: Pathogens. 2021 Apr 13;10(4):468. doi: 10.3390/pathogens10040468 (PMC8070065; doi:10.3390/pathogens10040468)
Supplement: Supplementary file 1 [file pathogens-10-00468-s001.zip › pathogens-1135369 supplementary/Figure-S2_REVISED.docx]

**SUPPLEMENTARY MATERIALS – FIGURE S2**

**Untargeted metabolomics insights of newborns with congenital Zika infection**

Estéfane da C. Nunes, Ana M. B. de Filippis on behalf of ZikAction Consortium, Taiane do E. S. Pereira, Nieli R. da C. Faria, Álvaro Salgado, Cleiton S. Santos, Teresa C. P. X. Carvalho, Juan I. Calcagno Flávia L. L. Chalhoub, David Brown, Marta Giovanetti, Luiz C. J. Alcantara, Fernanda Khouri Barreto, Isadora C. de Siqueira, and Gisele A. B. Canuto.


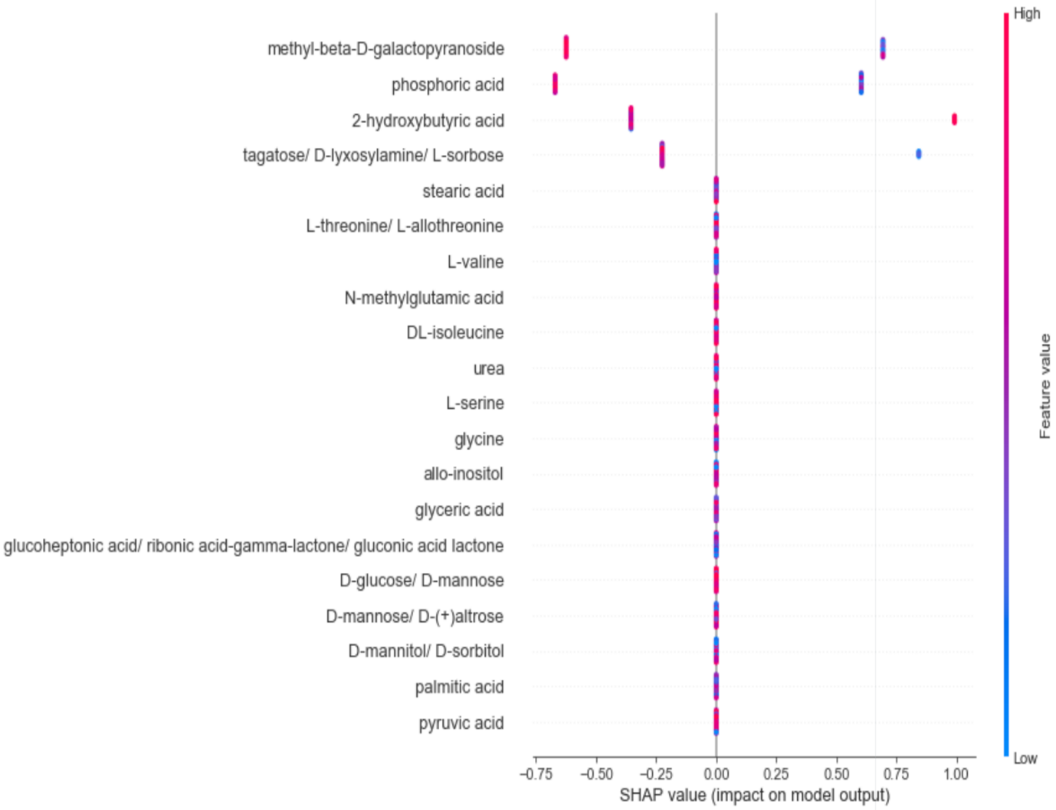

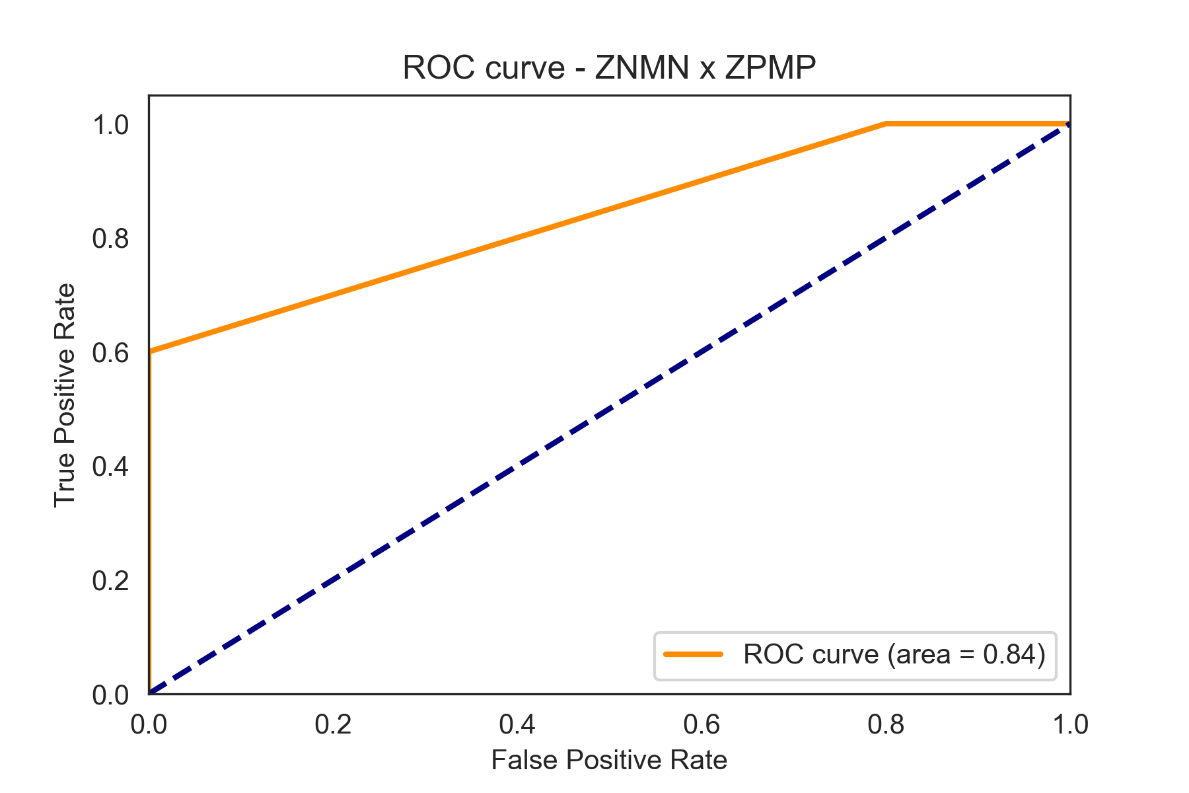


(a)


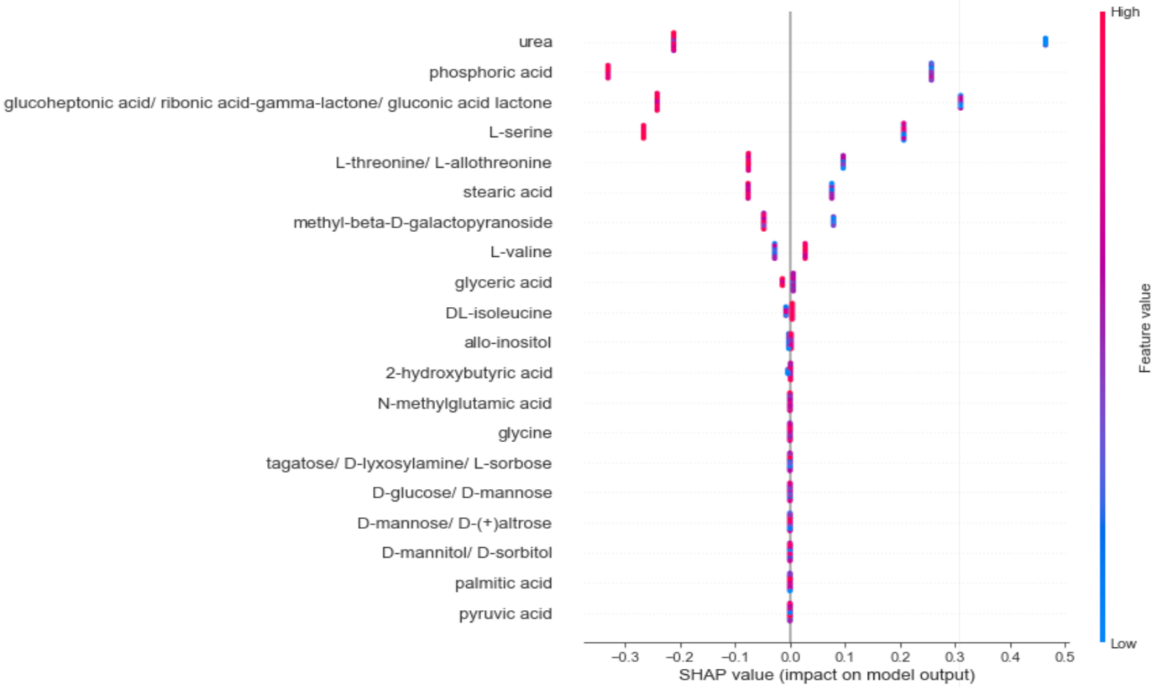

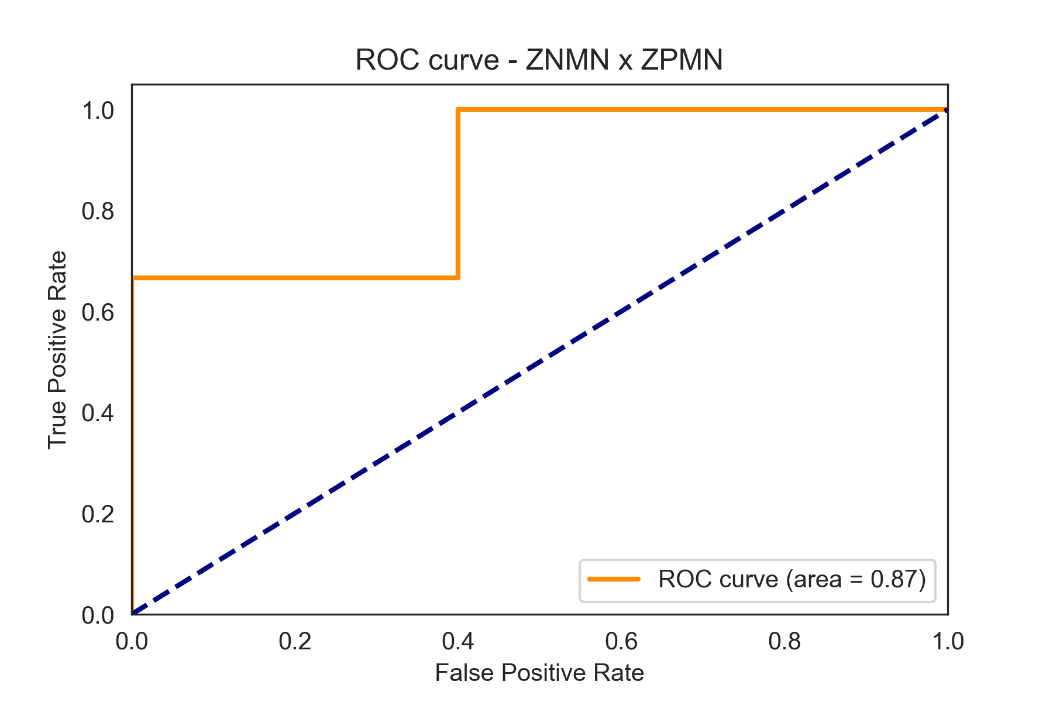


(b)


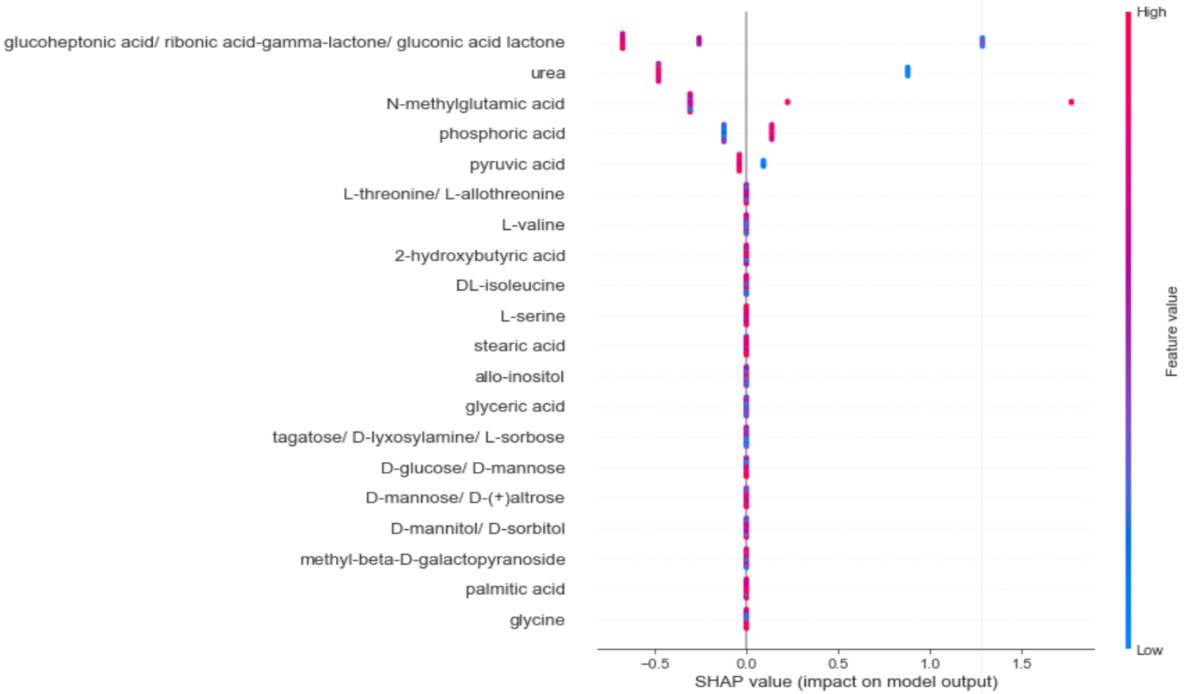

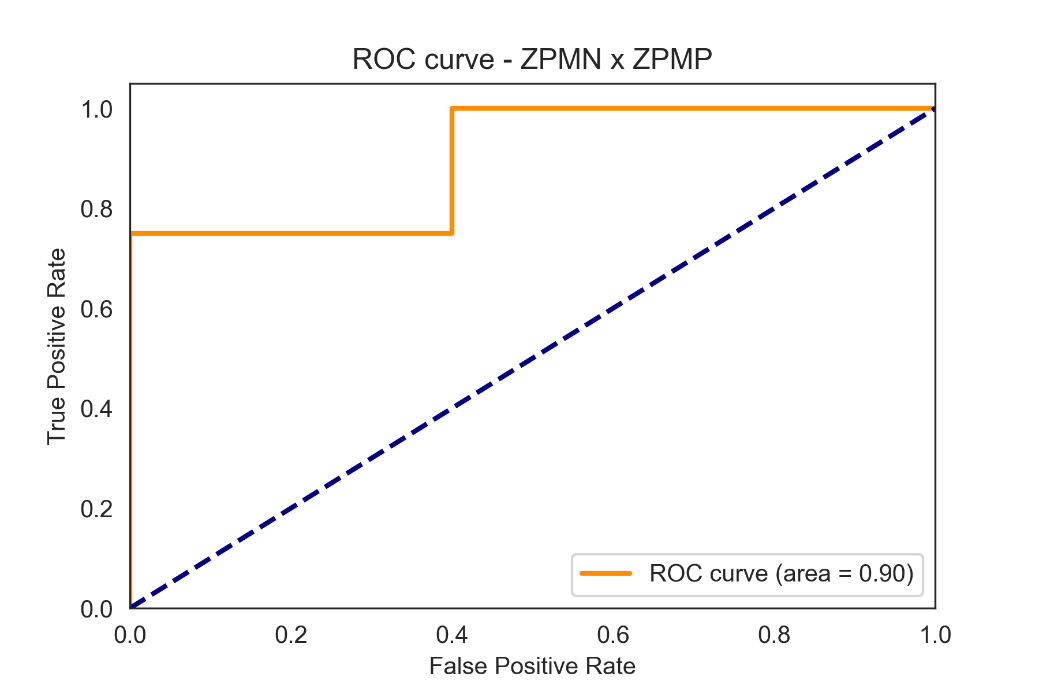


(c)

**Figure S2.** ZHAP analysis showing the influence of the metabolites for group classification and ROC curves attesting the performance of ML model. (**a**) ZPMP *vs.* ZNMN, (**b**) ZPMN *vs.* ZNMN, and (**c**) ZPMP *vs.* ZPMN. Group samples: ZPMP, zika virus with microcephaly; ZPMN, zika virus without microcephaly; ZNMN, control.
